# 479. Predisposing factors and outcomes of infections due to rare yeasts: a study from an apex medical center in India (2018-2020).

**DOI:** 10.1093/ofid/ofac492.537

**Published:** 2022-12-15

**Authors:** Gagandeep Singh, Immaculata Xess, Souradeep Chowdhury, Manish Soneja, Janya Sachdev, Azka Iram

**Affiliations:** All India Institute of Medical Sciences, New Delhi, New Delhi, Delhi, India; All India Institute of Medical Sciences, New Delhi, New Delhi, Delhi, India; All India Institute Of Medical Sciences, Kolkata, West Bengal, India; All India Institute Of Medical Sciences, Kolkata, West Bengal, India; All India Institute of Medical Sciences, New Delhi, New Delhi, Delhi, India; All India Institute of Medical Sciences, New Delhi, New Delhi, Delhi, India

## Abstract

**Background:**

Fungi are ubiquitous in the environment, and for patients with risk factors, they can present as pathogens. Unusual fungi often pose a diagnostic challenge and when encountered in practice, albeit infrequently, can delay diagnosis and treatment.

**Methods:**

Over a period of three years (2018-2020), such cases were followed up till death or discharge and all details were noted down. All patients were diagnosed to have invasive fungal disease according to the EORTC/MSG criteria. Species identification was performed using MALDI-TOF MS and antifungal susceptibility testing (AFST) was done using standard CLSI guidelines.

**Results:**

A total of 14 cases of rare yeasts were noted out of a total of 155 infections due to yeasts. Among these 5 presented with fungemia and 9 with invasive fungal infections (IFI). Of the IFI clinical isolates, there were 2 pus samples and 7 sterile fluids. The majority of these 14 patients were male (11) and belonged to extremes of age (8). The risk factors included CKD (2), prior history of tuberculosis (2), CLD (1), congenital heart disease (2), and type 2 diabetes mellitus (1). Pathogens isolated were *Candida guilliermondii* (2), *Candida pelliculosa* (2), *Lodderomyces elongisporus* (1) from the blood samples, and *Trichosporon asahii* (6), *Candida guilliermondii* (1), *Candida kefyr* (1), and *Geotrichum* spp. (1) from the other samples. Certain Candida species showed higher MICs to azoles and echinocandins. Trichosporon and Geotrichum showed higher MICs to azoles.

Unfortunately, half of the patients (7) received no antifungals. Of those who did, 4 received mono therapy with azoles or Amphotericin B, while other 3 received combination or sequential therapy with azoles, echinocandins and Amphotericin B. Of the 14 patients, 3 patients expired. The etiological agents in these cases were *Candida pelliculosa* (fungemia) and *Trichosporon asahii* (IFI, 2 cases).

**Conclusion:**

This study aims to highlight the clinical presentation of uncommon yeasts, presenting both as bloodstream infections (BSI) and as invasive disease. Improved methods of detection will help us improve our diagnostic accuracy of uncommon pathogens. The clinician should liaise closely with the laboratory, and treatment should be instituted according to the AFST data.

**Disclosures:**

**All Authors**: No reported disclosures.

